# Circulating Cell and Plasma microRNA Profiles Differ between Non-ST-Segment and ST-Segment-Elevation Myocardial Infarction

**DOI:** 10.4172/2327-4972.1000108

**Published:** 2013-07-10

**Authors:** Jeanine A Ward, Nada Esa, Rahul Pidikiti, Jane E Freedman, John F Keaney, Kahraman Tanriverdi, Olga Vitseva, Victor Ambros, Rosalind Lee, David D McManus

**Affiliations:** 1 Department of Emergency Medicine, University of Massachusetts Medical School, Worcester, MA, USA; 2 Cardiac Electrophysiology Section, Cardiovascular Medicine Division, Department of Medicine, University of Massachusetts Medical School, Worcester, MA, USA; 3 Department of Molecular Biology, University of Massachusetts Medical School, Worcester, MA USA; 4 Department of Quantitative Health Sciences, University of Massachusetts, Medical School, Worcester, MA, USA; 5 Department of Biomedical Engineering, Worcester Polytechnic Institute, Worcester, MA, USA

**Keywords:** Acute coronary syndrome, ST Elevation Myocardial Infarction (STEMI), Non-ST Elevation Myocardial Infarction (NSTEMI), MicroRNA, biomarkers

## Abstract

**Background:**

Differences in plasma and whole blood expression microRNAs (miRNAs) in patients with an acute coronary syndrome (ACS) have been determined in both in vitro and in vivo studies. Although most circulating miRNAs are located in the cellular components of whole blood, little is known about the miRNA profiles of whole blood subcomponents, including plasma, platelets and leukocytes in patients with myocardial ischemia.

**Methods:**

Thirteen patients with a ST-segment-elevation (STEMI) or non-ST-segment elevation (NSTEMI) myocardial infarction were identified in the University of Massachusetts Medical Center Emergency Department (ED) or cardiac catheterization laboratory between February and June of 2012. Whole blood was obtained from arterial blood samples at the time of cardiac catheterization and cell-specific miRNA profiling was performed. Expression of 343 miRNAs was quantified from whole blood, plasma, platelets, and peripheral blood mononuclear cells using a high-throughput, quantitative Real-Time polymerase-chain reaction system (qRT-PCR).

**Results:**

MiRNAs associated with STEMI as compared to NSTEMI patients included miR-25-3p, miR-221-3p, and miR-374b-5p. MiRNA 30d-5p was associated with plasma, platelets, and leukocytes in both STEMI and NSTEMI patients; miRNAs 221-3p and 483-5p were correlated with plasma and platelets only in NSTEMI patients.

**Conclusions:**

Cell-specific miRNA profiles differed between patients with STEMI and NSTEMI. The miRNA distribution is also unique amongst plasma, platelets, and leukocytes in patients with ischemic heart disease or ACS. Our findings suggest unique miRNA profiles among the circulating subcomponents in patients presenting with myocardial ischemia.

## Introduction

Up to 10 million Americans report to an Emergency Department (ED) for chest pain yearly and this number is expected to climb with continued aging of the United States (U.S.) population [1]. The substantial number of emergent hospitalizations for acute coronary syndrome (ACS), including ST-elevation myocardial infarction (STEMI), Non ST-elevation myocardial infarction (NSTEMI), and unstable angina (UA), represents a significant financial strain to both patients and the healthcare system [2,3]. This burden could be ameliorated, and more efficient treatment administered, if a more rapid, sensitive, and specific biomarker could differentiate between the spectrum of ACS (i.e. STEMI, NSTEMI, and UA) as well as differentiate from other less concerning causes of chest pain, such as gastroesophageal reflux (GERD) [4].

MiRNA profiles have been described from whole blood of patients with an acute coronary syndrome [5,6]. Although previous studies have noted miRNAs specifically in the plasma of patients with myocardial ischemia, additional information is required to further understand the miRNA species in whole blood subcomponents, such as plasma, platelets and leukocytes. However, by examining circulating blood subcomponents, important biomarkers unique to ACS may be identified. For example, miRNAs may be ideal biomarkers for accelerating the diagnosis of MI in emergency department patients [7,8].

Differences in miRNA expression patterns in coronary artery disease (CAD), acute coronary syndrome (ACS) and microparticles (MP) have been previously investigated in both in vitro and in vivo models [9]. Previous studies have demonstrated differences in inflammatory kinetics between STEMI and NSTEMI patients [10]. However, little else is known about the miRNA profiles of circulating blood pools, platelets, and lymphocytes in patients within STEMI and NSTEMI. Therefore, the goal of this study was twofold: 1) to investigate if miRNA expression differed between patients presenting with STEMI and NSTEMI in a Central Massachusetts academic setting and 2) to describe the miRNA expression profiles of patients with an ACS further stratified by circulating subcomponents, including plasma, platelets, and leukocytes.

## Materials and Methods

### Participant recruitment

102 patients presenting with an ACS were identified in the University of Massachusetts Medical Center ED or cardiac catheterization laboratory between February and June of 2012. Of the 102 patients screened for inclusion, 13 fulfilled study inclusion criteria for myocardial infarction, including both NSTEMI and STEMI [11,12]. Delayed, written informed consent was obtained from all enrollees after they were clinically stabilized. This study was approved by the University of Massachusetts Institutional Review Board (Docket H:14125).

### Sample acquisition and handling

All participants underwent phlebotomy at the time of their cardiac catheterization procedure. 10 milliliters of arterial blood was obtained from a radial or femoral access site and transferred in a sterile fashion into PAXgene (Qiagen, CA), CPT (Becton-Dickenson, NJ), and ethylenediaminetetraacetic acid (EDTA) tubes. Samples were placed immediately on ice and then taken within 10 minutes to the study laboratory.

### RNA isolation

Total RNA including small RNA and miRNAs isolated from plasma samples by using miRNeasy kit (Qiagen Valencia, CA) 100 (µl plasma and 700 µl RLT solution mixed and incubated at room temperature for 10 minutes then isolation performed according to the protocol provided with the kit. Isolated RNA samples were stored at −80°C until cDNA reaction.

### Reverse transcription reaction

Isolated RNAs were reverse-transcribed into cDNA in 5 (µl final reaction volumes using TaqMan MicroRNA Reverse Transcription Kit (Applied Biosystems, Foster City, CA, USA). All reactions were performed as specified in the manufacturers protocol (except modification in final volume): 2 (µl total RNA were added to 3 µl of the RT reaction mix (MegaPlex RT Primers 10X, dNTPs with dTTP 100mM, MultiScribe Reverse Transcriptase 50 U/µl, 10X RT Buffer, MgCl_2_ 25 mM, RNase Inhibitor 20 U/µl and Nuclease-free water). Reverse transcription was performed using 384 well Thermal Cycler (Veriti 384, Applied Biosystems, Foster City, CA, USA). 343 cardiac specific microRNAs were selected as previously described (The SABRe CVD Initiative; dbGaP Study Accession: phs000007.vl8.p7). Reaction conditions were: 16°C for 120 sec, 42°C for 60 sec, 50°C for 1 sec, and these three steps repeated for 40 cycles and 85°C for 300 sec and 4°C for hold until further processing or storage. cDNA samples were kept at −80°C until Real-Time PCR analysis.

### Preamplification

A preamplification reaction was performed after the reverse transcription using the TaqMan PreAmp Master Mix 2X (Applied Biosystems, Foster City, CA, USA) as well as the MegaPlex Human PreAmp Primer Pools Set v3.0 (Applied Biosystems, Foster City, CA, USA). All reactions were performed as specified in the protocols of the manufacturer. Two (µl of 1:5 diluted Reverse Transcription products were added to 3 µl of the PreAmplification mixture. The reaction volume was 5 µl miRNA TaqMan PreAmp Thermal Protocol was performed using 384 well Thermal Cycler (Veriti 384, Applied Biosystems, Foster City, CA, USA) as follows: 95°C for 600 sec, 55°C for 120 sec and 72°C for 120 sec, followed by 18 cycles with 95°C for 15 sec, 60°C for 240 sec, finally 600 sec at 99.9°C; and hold at 4°C.

### Real-Time PCR

Real-Time PCR reactions (qRT- PCR) were performed using the high-throughput BioMark Real-Time PCR system (Fluidigm, South San Francisco, CA). PreAmplified cDNA samples were diluted with DNA Suspension buffer (Teknova, Hollister, CA, USA) 1:5 times. Four hundred ninety (µl of TaqMan Universal PCR Master Mix, No AmpErase UNG, (Applied Biosystems, Foster City, CA, USA), and 49 µl of 20x GE Sample Loading Reagent (Fluidigm, South San Francisco, CA) mixed and pipetted into a 96 well plate as 3.85 (µl. One: 10 times diluted 3.15 µl of PreAmplified cDNA samples pipetted into each well and mixed. Then 5 µl of this mixture pipetted into sample inlets of a 96.96 Dynamic Arrays (Fluidigm, San Francisco, USA) 4.0 µl 1:1 diluted 20X TaqMan miRNA Assays pipetted into assay inlets of a 96.96 Dynamic array (Fluidigm). The BioMark IFC controller HX (Fluidigm, San Francisco, CA) was used to distribute the assay mix and sample mix from the loading inlets into the 96.96 Dynamic array reaction chambers for qRT-PCR by Fluidigm’s Integrated Fluidic Circuit Technology. Real-Time PCR was performed by using BioMark System by using following protocol; Hold on at 95°C for 600 sec. and 95°C for 15 sec, 60°C for 60 sec for 30 cycles.

### Statistical analysis

All statistical analyses were performed using the Biogazelle qbasePLUS 2.0 software. Mann-Whitney U test used and p<0.05 was considered statistically significant. Gene targets of miRNAs found to be up or down-regulated in patients with STEMI as compared with NSTEMI were predicted using the following algorithms: miRDB (miRDB.org) [13] and MirWalk (http://www.umm.uni-heidelberg.de/apps/zmf/mirwalk) [14].

## Results

### Patient demographics

As shown in Table 1, most participants with STEMI (N=9) were male (67%) and middle aged (58.4 ± 3.8 years). Participants presenting with NSTEMI in our cohort (N=4) were uniformly male and were slightly older (68.5 ± 5.3 years) than participants with STEMI. The majority of participants in both STEMI and NSTEMI groups had a history of hypertension (78% and 75%, respectively) and a minority had a history of diabetes (11% and 25%, respectively). All of the participants were Caucasian in race origin. A majority of the NSTEMI patients as compared to STEMI patients were receiving outpatient treatment with a statin, beta-blocker, ACE inhibitor, aspirin and clopidogrel prior to hospitalization. There were essentially no differences in the leukocyte, hemoglobin/hematocrit, and platelet counts between these two cohorts (Table 1).

### Cardiac catheterization results

The primary culprit lesion at the time of cardiac catheterization for 5 of the patients (all STEMI) was the mid-left anterior descending (LAD) coronary artery. The right coronary artery (RCA) was the culprit vessel in 3 patients (all STEMI). The obtuse marginal and 2^nd^ diagonals were involved in 2 patients (NSTEMI) patients. The culprit lesion could not be determined in 2 patients with diffuse, three-vessel coronary disease, both NSTEMI (Table 2). An intracoronary (IC) thrombectomy was performed for documented thrombus in 9 patients. The peak troponin levels for STEMI and NSTEMI patients were 168.9 and 75.8 (± SD needed), respectively (Table 2).

### MiRNAs in STEMI vs. NSTEMI: Plasma, Platelets, Peripheral Blood Mononuclear Cells (PBMCs)

**Figure 1:**
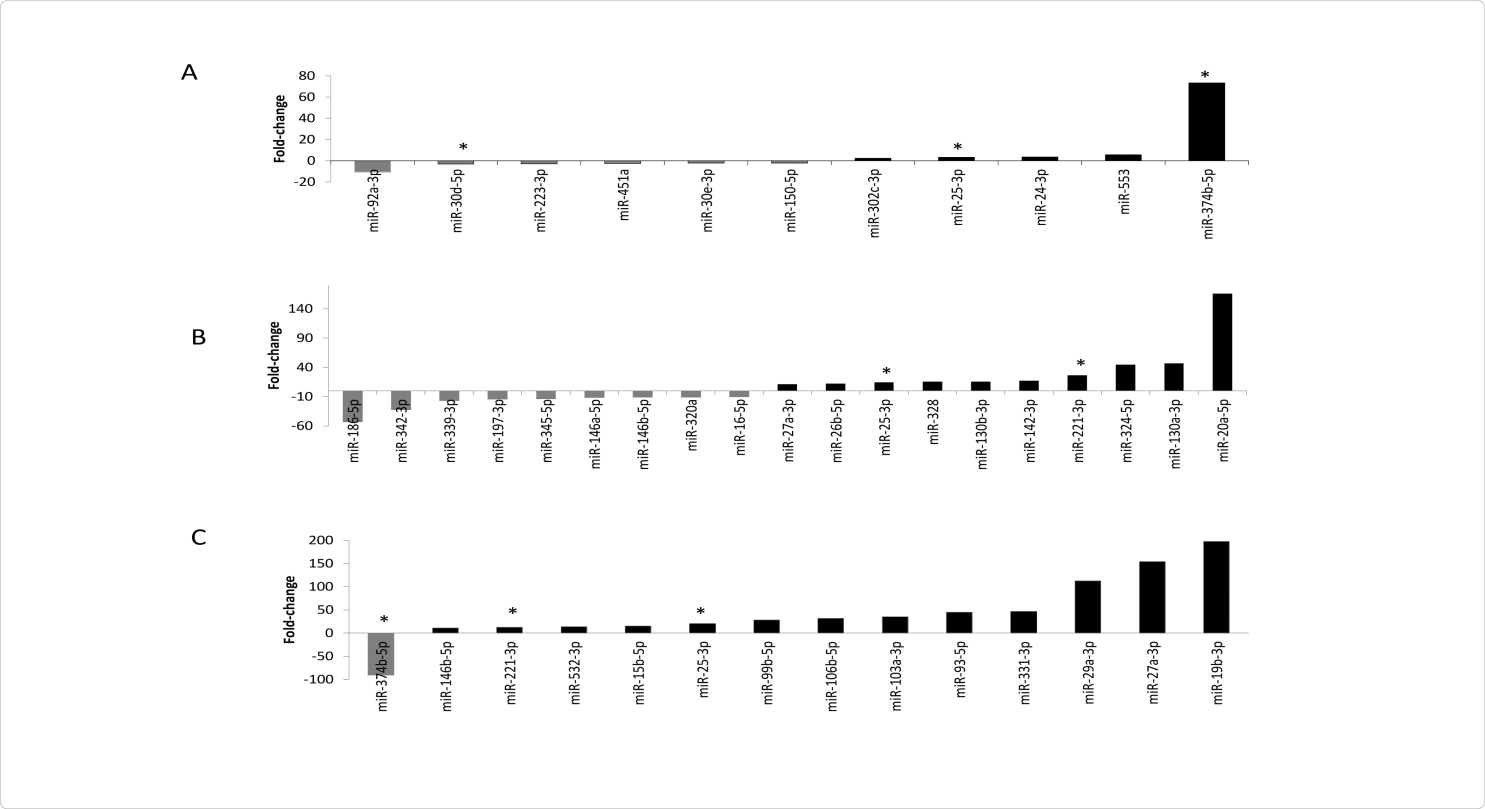
MiRNA Levels in STEMI vs. NSTEMI: Plasma, Platelets, Peripheral Blood Mononuclear Cells (PBMCs).

In an effort to better understand differences between circulating miRNA profiles further stratified in patients with STEMI and NSTEMI, we found that expression of plasma miRNAs 92a-3p and 30d-5p, platelet miRNAs 186-5p and 342-3p, and *PBMCs* miRNAs 374b-5p were significantly lower in patients with STEMI as compared with NSTEMI (Figures 1A-1C). In contrast, plasma miRNAs 25-3p, and 374b-5p, platelet miRNAs 25-3p and 221-3p, and *PBMCs* miRNAs 25-3p and 221-3p, were significantly higher in patients with STEMI than NSTEMI (Figures 1A-1C).

**Figure 2:**
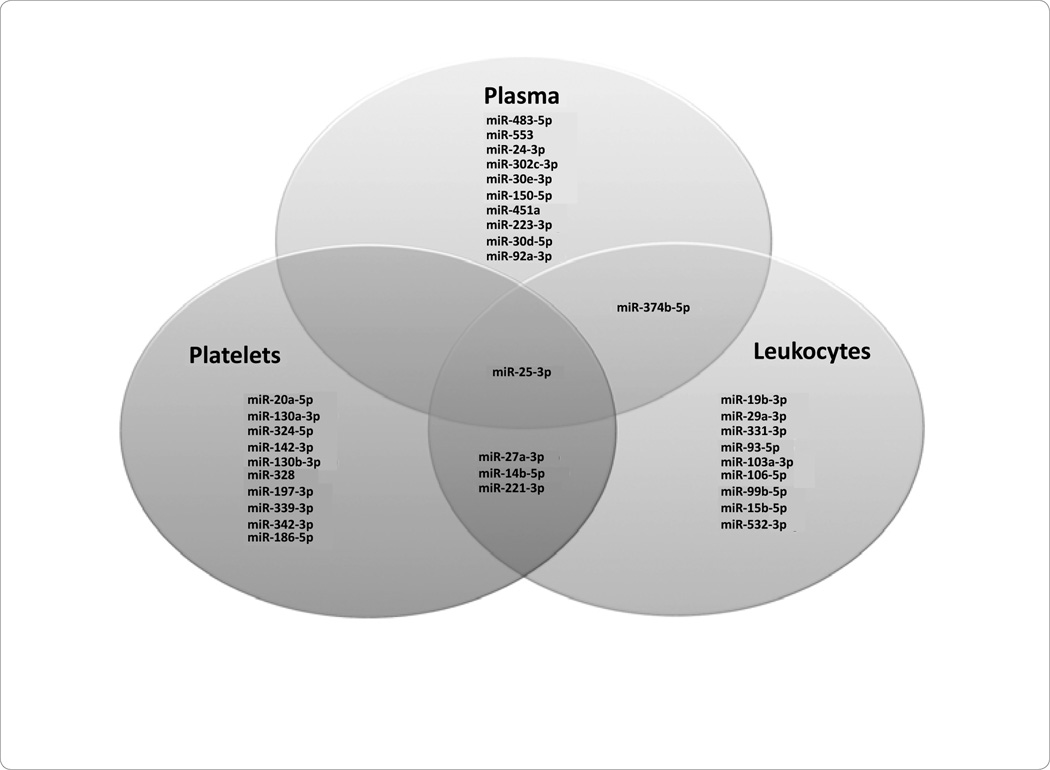
Common miRNAs in STEMI vs. NSTEMI: Plasma, Platelets, Peripheral Blood Mononuclear Cells (PBMCs).

In STEMI patients as compared to NSTEMI patients, there were distinct miRNAs identified, uniquely correlated to the subcomponents investigated. Namely, miRNA 25-3p was characterized associated with plasma, platelets, and *PBMCs* (Figure 2). However, miRNA 27a-3p 146b-5p, and 221-3p were found in platelets and leukocytes only; miRNA 374-5p was identified in plasma and *PBMCs* only (Figure 2).

### STEMI MicroRNAs in Plasma, Platelets, and PBMCs

**Figure 3:**
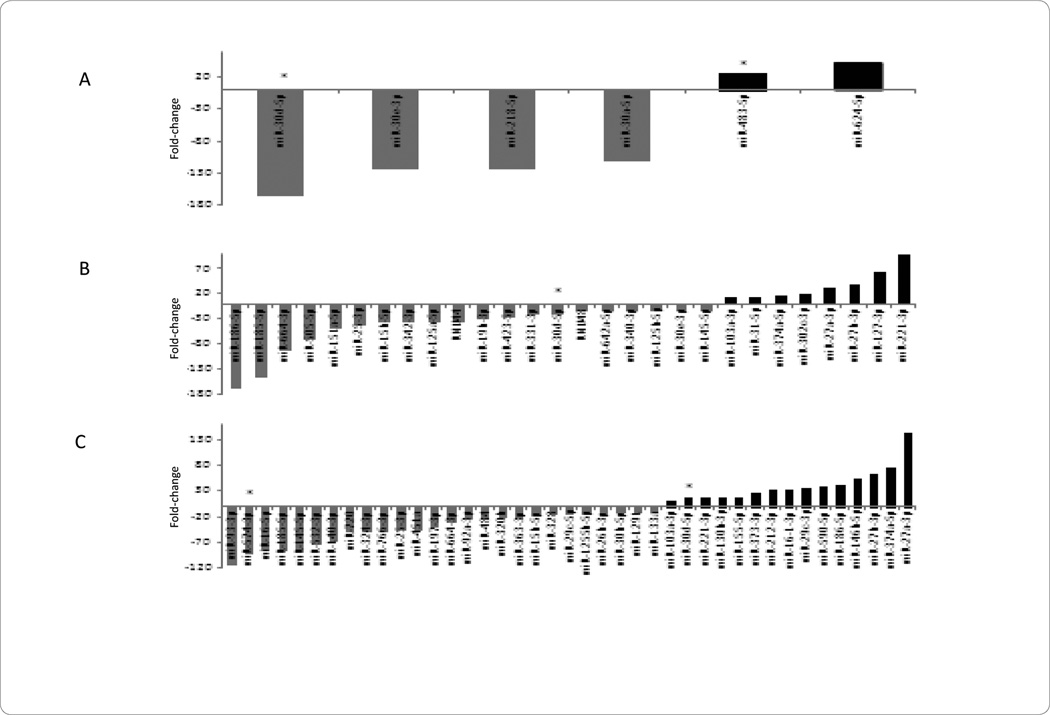
STEMI MicroRNA Levels in Plasma, Platelets, and Peripheral Blood Mononuclear Cells (PBMCs).

**Figure 4:**
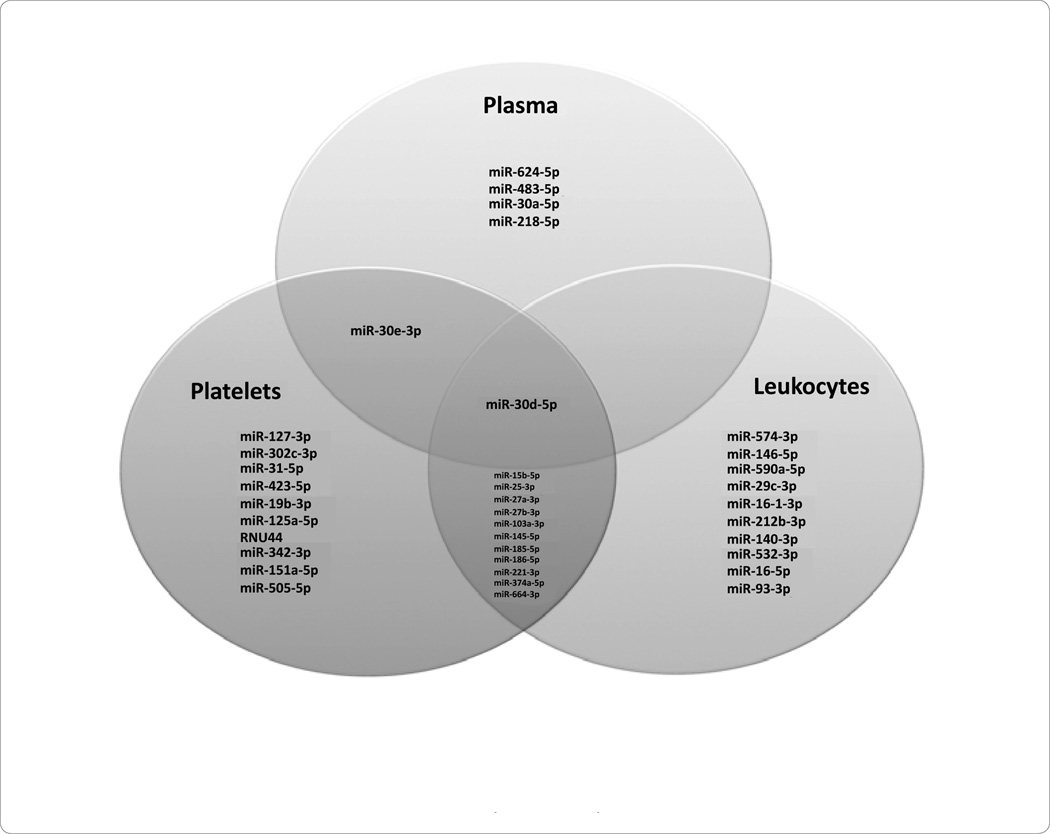
STEMI patients: Common miRNAs in Plasma, Platelets, and Peripheral Blood Mononuclear Cells (PBMCs).

In the plasma of patients presenting with STEMI, the most downregulated miRNAs included 30d-5p and 30e-3p, yet the most upregulated miRNAs included 483-5p and 624-5p (Figure 3A). In the platelets of patients presenting with STEMI, the most downregulated miRNAs included 186-5p and 185-5p; miRNAs 127-3p and 221-3p were upregulated in this cellular subcomponent (Figure 3B). The *PBMCs* of patients presenting with STEMI demonstrated downregulation of miRNAs 93-3p and 574-3p; the most upregulated miRNAs in this subcomponent included 374a-5p and 27a-3p (Figure 3C). Common miRNAs in STEMI patients include 30d-5p in plasma, platelets, and *PBMCs*, 30e-3p between plasma and platelets, and 15b-5p, 25-3p and 27a-3p between platelets and *PBMCs* (Figure 4).

### NSTEMI MicroRNAs in Plasma, Platelets, and PBMCs

**Figure 5:**
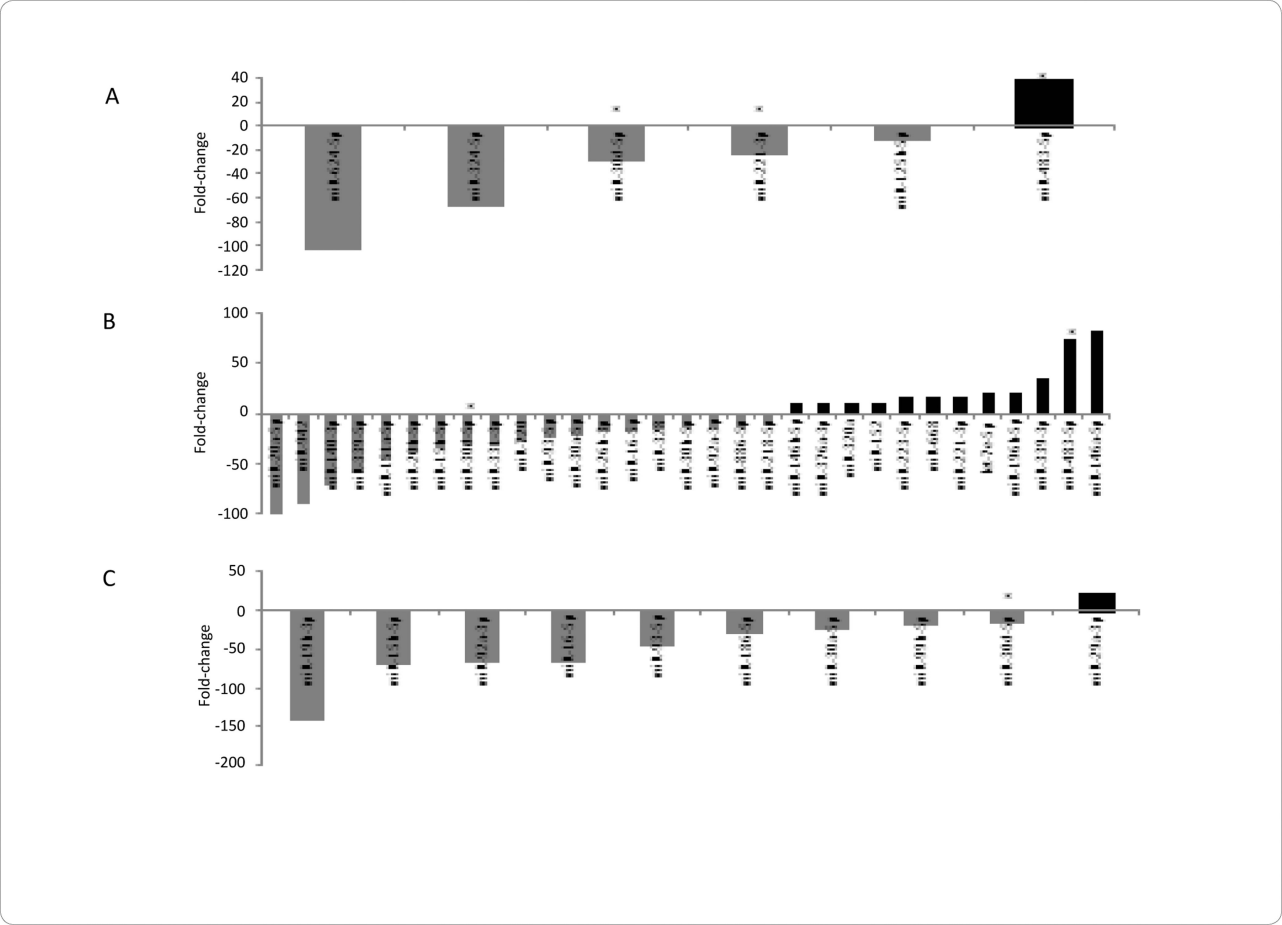
NSTEMI MicroRNA Levels in Plasma, Platelets, and Peripheral Blood Mononuclear Cells (PBMCs).

**Figure 6:**
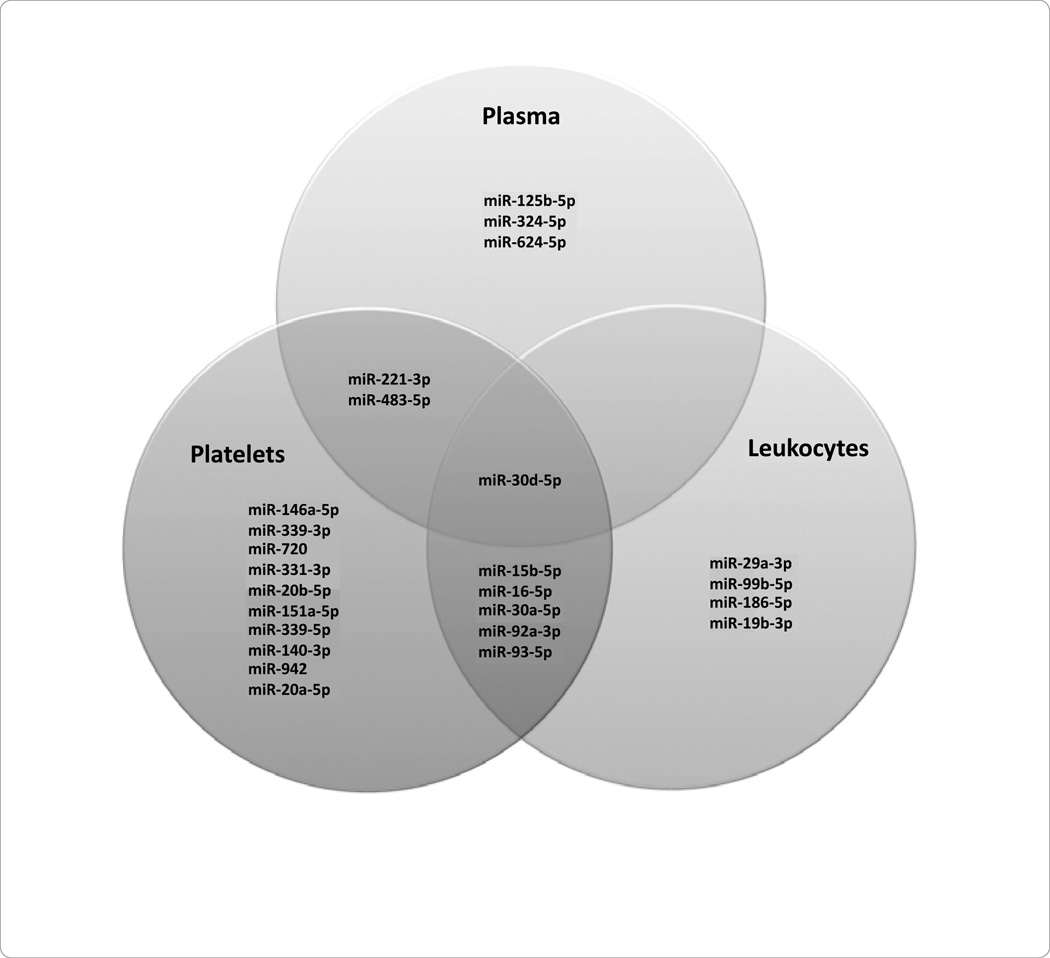
NSTEMI patients: Common miRNAs in Plasma, Platelets, and Peripheral Blood Mononuclear Cells (PBMCs).

In the plasma of patients presenting with NSTEMI the most downregulated miRNAs included 624-5p and 324-5p; the most upregulated miRNAs included 483-5p (Figure 5A). In patients presenting with NSTEMI, the most downregulated miRNAs in platelets were 20a-5p and 942, while the most upregulated miRNAs included 483-5p and 146a-5p (Figure 5B). In the *PBMCs* of patients presenting with NSTEMI, the most downregulated miRNAs included 19b-3p and 15b-5p; the most upregulated miRNAs consisted of 29a-3p (Figure 5C). MiRNA 30d-5p was found in all of the subcomponents of NSTEMI patients. MiRNA 221-3p and 483-5p was associated with both the plasma and platelet subcomponents of NSTEMI patients; miRNA 15b-5p, 16-5p, 30a-5p were common between platelets and *PBMCs* in NSTEMI patients (Figure 6).

## Discussion

In this exploratory analysis of 13 patients with ACS, we employed novel high-throughput methods to quantify expression of 343 miRNAs from distinct circulating blood pools. We found that 5 miRNAs were differentially expressed across plasma, platelets, and *PBMCs* in patients with NSTEMI and STEMI, including several miRNAs implicated in regulation of processes important to the pathogenesis of ACS [15–18].

### MicroRNA profiles of patients with STEMI compared to NSTEMI

MiRNAs 25-3p and 221-3p were both found upregulated in STEMI compared to NSTEMI patients (Figure 1 and Figure 2). Of interest, previously identified validated targets for miRNA 25-3p and 221-3p include CDKN1C (or p57/kip2) [19]. CDKN1C, a cell cycle inhibitor, was previously found associated with apoptosis, transcriptional regulation, and cell migration [20]. Of interest, Galardi et al. determined knockdown of miRNA 221-3p via antisense LNA oligonucleotides in a prostate carcinoma model reduced clonogenicity *in vitro*, supporting the role of miRNA 221-3p inhibiting CDKN1C and subsequent G1/S cell cycle progression [21]. Therefore, a role of miRNA 25-3p and 221-3p is possible in also activating CDK2 vis CDKN1C inhibition in the setting of STEMI, where cell proliferation is necessary in the milieu of acute myocyte damage and cell death [19].

Since it is well known multiple miRNAs may function at the same target, it is possible miRNA 25-3p and 221-3p are functioning in conjunction, both binding to the CDN1C 3’UTR to enhance CDK2 activation and cell cycle progression through the G2/S phase. Although cell activation and proliferation are necessary in the setting of myocyte death in the setting of STEMI, such mechanisms would also presumably be important in the setting of NSTEMI. How and why this mechanism is unique as compared to NSTEMI patients, which would also presumably require cell proliferation in the myocyte damage still needs to be elucidated.

MiRNA 374b-5p was significantly up-regulated in plasma, yet down-regulated in *PBMCs* in patients with STEMI relative to patients with NSTEMI (Figure 1). Predictive miRNA target software suggests that the targets of miRNA 374b-5p include cell adhesion molecule 2 (CAD2) and fibroblast growth factor 5 (FGF-5) [13]. Of note, CAD2 has been implicated in coronary artery disease and patient death; FGF-5 has been associated with ischemic heart disease, in vitro [15,16,22]. With the targets of miRNA 375-5p, namely CAD2 and FDF-5, being already demonstrated involved with heart disease, suggests a possible involvement of miR-374b-5p in myocardial infarction [15,16].

### STEMI only-plasma, platelets, and leukocytes

In patients with STEMI, miRNA 30d-5p was downregulated in plasma and platelets, but upregulated in the *PBMCs* (Figure 3). Validated miRNA 30d-5p targets include G-protein alpha inhibiting activity polypeptide 2 (GNAI2) or Galphai2, an alpha subunit of G proteins involved in adenylate cyclase activation and intracellular signaling [23]. MiRNA 30d-5p was shown to bind GNAI2 in a hepatocellular carcinoma (HCC) via luciferase reporter assay technology, where miRNA 30d-5p associated GAI2 knockdown increased HCC cell migration and invasion [24]. Of note, absence of Galphai2 has been shown associated with ventricular arrhythmias in a Galphai2 knockout mice model when challenged with programmed electrical stimulation. A potential role of miRNA 30d-5p, Galphai2, and STEMI is possible, but future studies would be needed to elucidate this possible relationship [25,26].

MiRNA 483-5p was found upregulated in plasma of STEMI patients (Figure 3A). Interestingly, miRNA 483-5p, transcribed from the intronic region of insulin-like growth factor-2, was recently found to inhibit angiogenesis, potentially by binding to serum response factor (SRF) [17]. Of note, overexpression of miRNA 483-5p was shown to inhibit angiogenesis, while inhibition of miRNA 483-5p can promote angiogenesis, both *in vitro* [17]. In addition, miRNA 483-5p was shown downregulated in human umbilical vein endothelial cells (HUVECS) cells under hypoxic conditions, again implicating a role in angiogenesis [17].

### NSTEMI only-plasma, platelets, and PBMCs

In addition to miRNA 30d-5p association in STEMI patients, it was also shown downregulated in the plasma, platelets, and *PBMCs* of NSTEMI patients (Figure 5). Although, miRNA 30d-5p can bind to alpha subunits of G-proteins [24], it has also been shown to bind to p53, a tumor suppressor gene involved in tumorigenesis and cell cycle progression [27]. MiRNA 30d-5p was shown to bind to the 3’UTR of the p53 transcript, thereby reducing p53 protein expression and the transcription of associated p53 related genes in a multiple myeloma model [28]. Conversely, inhibition of miRNA 30d-5p increased p53 protein expression and corresponding expected apoptosis [28]. Of note, p53 is known to be involved in atherosclerotic plaque disruption, where overexpression of p53 in adenoviral constructs can induce plaque rupture in a rabbit model [18]. Therefore, the role of miRNA 30d-5p, p53, and possible role in NSTEMI is evident.

In addition to being upregulated in STEMI compared to NSTEMI patients (Figure 1), miRNA 221-3p was also shown upregulated in plasma, but downregulated in platelets of NSTEMI patients (Figure 5). Why miRNA 221-3p was downregulated in platelets of NSTEMI patients, yet the clinical significance is currently unclear. What has been demonstrated is miRNA 221-3p also has similar targets as miRNA 25-3p, again including the cell cycle inhibitor CDKN1C. MiRNA 221-3p binding to CDKN1C, demonstrated in glioblastoma [29] and thyroid papillary carcinomas [30] cells, respectively, may also play a role in cell cycle progression in the setting of myocyte proliferation in the setting of myocardial ischemia.

As previously mentioned, miRNA 483-5p was found upregulated in the plasma of STEMI patients compared to whole blood. MiRNA 483-5p was also found up-regulated in both the plasma and platelets of NSTEMI patients (Figure 5A and 5B). To have miRNA 483-5p upregulated in the plasma of STEMI patients (Figure 3A), as well as in both the plasma and platelets in the NSTEMI patients (Figure 5A and 5B) is perplexing since angiogenesis would presumably be initiated in the setting of hypoxia. Possible explanations include additional targets and roles for 483-5p other than those related to angiogenesis. Also, with ischemia being a dynamic process and the timing of plasma procurement differing amongst patients, a more diverse role for miRNA 483-5p may be possible.

MiRNAs are rapidly emerging as biomarkers for acute myocardial infarction [22,31,32]. A study of 33 patients with acute STEMI found increased circulating plasma levels of miRNA 1, 133a, 133b, and 499-, and decreased levels of plasma miRNA 122 and 375 [31]. MiRNAs 1291 and 663b have been used to discriminate patients with STEMIs from control cases with particularly high sensitivity, specificity and accuracy [6]. Myocyte specific miRNA 208b and 499 were detectable in patients with cardiac damage, including acute myocardial infarction [32]. MiRNA-21 has been associated with interstitial fibrosis and cardiac hypertrophy and miRNA-320 with cardiac ischemia and reperfusion injury, respectively [33,34]. MiRNA-133a and miRNA-208b are associated with increased risk of death in patients presenting with acute coronary syndrome [35]. Finally, miRNA-133a and miRNA-499 have been shown released from the heart via trans coronary circulation in troponin-positive patients [36,37]. These miRNAs were not found either up or downregulated in our study. This may be due to differences in either the testing strategies employed, timing of samples obtained, or in the patient populations evaluated. Interestingly, as related to miRNA kinetics over time, Liebetrau et al. [38] recently determined increased circulating levels of mirR-1, miR-208a, and miR-133a over a 4 hour time frame after AMI induction [38]. Vogel et al. [39] revealed changes in miR 1915 and miR-181c over time in 18 troponin negative STEMI patients [39]. Both of these studies continue to support the ever growing body of literature supporting the role miRNAs as potential circulating biomarkers for myocardial damage [31,32,35]. Another advantage of employing miRNA as a circulating biomarker of AMI is phlebotimized blood draws are routine and with minimal adverse effects as compared to the increased risk of hemorrhage and sedation medication concerns as compared to coronary catheterization procedures. Finally and of note, literature has revealed difficulty in miRNA identification in patients treated with heparin previous to miRNA quantification; none of the patients in our study were treated with heparin prior to sample procurement [40].

### Implications

This proposal represents a significant advance, demonstrating unique miRNA profiles between the plasma, platelets, and leukocytes across both STEMI and NSTEMI patients (Figure 2). Our findings have important implications since the miRNAs found to be upregulated both in participants with STEMI and NSTEMI, namely miR-25-3p, miR-30d-5p, miR-221-3p, miR-374b-5p, and miR-483-5p) may be important to gene regulation across ACS subtypes. Our findings may also help to elucidate possible cross-talk occurring between plasma and cell types in the setting of ACS, also potentially uncovering potential therapeutic treatment targets. Our results may also be useful as additional miRNAs circulating biomarkers for ACS in addition to those already identified [35–39]. Our observation that miRNA profiles differ significantly across circulation subcomponents in the setting of ACS emphasize that future studies should consider subcomponent-specific miRNA expression analyses.

### Limitations

We recognize that our small sample size limits generalizability given the complexity of miRNAs within the biological system in addition to the diverse genetic, social, and treatment characteristics of patients presenting with an ACS. We are also aware the differences in patient demographics may also have affect these study results. Future studies including larger and more diverse patient populations will be needed to validate our hypothesis-generating findings. We are also aware that it would be ideal to have better information about the timing of symptom onset relative to miRNA quantification. The specific time of this upregulation still needs to be further evaluated. Unfortunately, little information is available with respect to pre-hospital delay in presentation or duration of symptoms, limiting our ability to precisely identify miRNA dysregulation with respect to symptom onset Future studies will include evaluating circulating miRNA profiles in well-defined patient populations and at different time points during the peri-infarct period as well as miRNA profiles associated with unstable angina and non-ACS associated causes of chest pain, such as GERD.

## Conclusion

This investigation reveals unique miRNA profiles in plasma, platelets, and *PBMCs* of STEMI and NSTEMI patients, including miRNA 25-3p, 30d-5p, 221-3p, 374b-5p, and 483-5p. This novel discovery suggests both that there may by unique, circulating subcomponent miRNAs that may allow for future differentiation of patients presenting with an ACS. We anticipate in future studies with larger numbers of patients, concomitant changes in other ACS associated miRNAs, including miRNA-1, miRNA-133, and miRNA-208, 483-5p and 601 in the setting of STEMI and NSTEMI (Table 3) [5,31,32]. We also anticipate future research will reveal unique miRNA profiles in circulating subcomponents of other aspects of an ACS, namely UA, in addition to non-ACS causes of chest pain.

## Figures and Tables

**Table 1:** Patient demographic characteristics

	STEMI (N=9)	NSTEMI (N=4)
**Demographics:**		
Mean age (years)	58.4 ±3.8	68.5 ± 5.33
Sex (Male)	6(67)	4(100)
Race-White	9(100)	4(100)
Heart Rate (BPM)	84.9 ±7.5	75.8 ±11.7
Blood Pressure (SBP/DBP)	151 ±10.5/91.6 ±12.5	137.5 ±4/81.3 ±3.2
Troponin	59.6 ±18.4	25.8 ±16.8
Creatinine	1.1 ±0.18	1.3 ±0.35
Leukocytes	10.9 ± 1.1	10.7± 1.5
Platelets	220.8 ±19.6	227.5 ±22.6
Hematocrit	40.5 ±1.8	39 ± 0.64
Hemoglobin	13.6 ±0.6	12.8 ±0.35
Body Mass Index (kg/m^2^), mean +/− SE	28.1 ±0.8	26.2 ±1.74
**Comorbid conditions:**		
Hypertension	7(78)	3(75)
Coronary artery disease	4(44)	2(50)
Diabetes Mellitus	1 (11)	1(25)
Dyslipidemia	4(44)	4(100)
Current smoking	5(56)	1(25)
Alcohol	2(22)	1(25)
Prior History PCI	4(44)	2(50)
Prior History CABG	0(0)	1(25)
**Pre-Hospital Medications:**		
Statins	2(22)	4(100)
Beta blocker	1 (11)	2(50)
Angiotensin-converting enzyme inhibitor /Angiotensin receptor blocker	3(33)	2(50)
Calcium channel blocker	2(22)	0(0)
Diuretics	2(22)	0(0)
Aspirin	3(33)	3(75)
Clopidogrel	1 (11)	3(75)
Nitroglycerin	3(33)	2(50)

ACE-I – Angiotensin Converting Enzyme Inhibitor; ARB – Angiotensin II Receptor Blocker

**Table 2:** Patient cardiac catheterization results

Subject #	ACS Type	Location of Culprit Lesion	Intra-coronary thrombus documented	Single or Multi vessel Disease**	EF (%)	Troponin (Initial)	Troponin (Peak)	Day of Cath relative to admission day (Day 0)
1	NSTEMI	Obtuse Marginal	Yes	Single	50	0.65	75.80	0
2	STEMI	RCA	No	Multi	65	22.83	73.50	0
3	STEMI	RCA	Yes	Single	45	42.67	42.67	0
4	STEMI	Mid LAD	Yes	Single	20	93.42	168.90	0
5	STEMI	Mid LAD	Yes	Single	30	1.83	89.60	0
6	STEMI	Proximal LAD	Yes	Single	30	0.06	16.85	0
7	STEMI	Mid LAD	Yes	Single	45	0.82	108.86	0
8	NSTEMI	Undetermined	No	Multi	35	0.01	13.96	1
9	NSTEMI	Undetermined	No	Multi	20	2.88	4.3	1
10	STEMI	Mid LAD	Yes	Single	35	1.66	8.47	0
11	STEMI	Mid LAD	Yes	Single	25	0.04	19.24	0
12	STEMI	Mid RCA	Yes	Multi	60	3.71	8.64	0
13	NSTEMI	2^nd^ Diagonal	Yes	Single	UTO	5.3	9.07	1

ACS – Acute Coronary Syndrome; IC – Intracoronary; EF – Ejection Fraction; NSTEMI – Non ST Segment Elevation Myocardial Infarction; STEMI – ST Segment Elevation Myocardial Infarction; RCA – Right Coronary Artery; LAD – Left Anterior Descending Coronary Artery; UTO – Unable To Obtain

**Table 3:** miRNAs Up or down-regulated in patients with an acute coronary syndrome and their target molecules^3^

MicroRNA	Target Gene	Resultant Effects	Ref
Mir-1	Hand 2	Reduction of ventricular myocytes; STEMI	[38]
Mir-30c	Increased CTGF	Cardiac hypertrophy; STEMI	[5]
Mir-126	Vascular endothelial growth factor (VEGF)	Heart valve development	[39]
Mir-133	Kv4-encoded fast potassium channel	QT prolongation; reduced myocardial fibrosis; STEMI	[40]
Mir-208	Myh7	Cardiac remodeling; diastolic dysfunction	[41]
Mir-320	Heat Shock Protein-20	Cardiac Ischemia/ Reperfusion Injury	[34]
Mir-374-5p	Cell Adhesion Molecule 2	Coronary Artery Disease	[41], [43], [44]
Mir-483-5p	Myomesin (MYOM2)	Interconnects major sarcomere structure	[27], [43]
Mir-574-3p	Coiled-coil domain family 1	Myocardial infarction	[41], [46]
Mir-499	Repressed histone deacetylase-4; Sox6	Enhanced cardiomyocyte differentiation; STEMI	[42]
Mir-601	TPM3	Tropomyosin 3	[27]
Mir-1275	Synaptotagmin VII	HUVEC cell shear stress	[11], [41]
Mir-1291	Matrix metallopeptidase-24	STEMI	[5]

* Using MirWalk (http://www.ma.uni-heidelberg.de/apps/zmf/mirwalk/) [14] Mirdb (http://www.mirdb.org) [13]
